# Nulliparas at Term with Premature Rupture of Membranes and an Unfavorable Cervix: Labor Induction with Prostaglandin or Oxytocin? A Retrospective Matched Case Study

**DOI:** 10.3390/jcm13123384

**Published:** 2024-06-09

**Authors:** Maayan Bas Lando, Ewida Majida, Amy Solnica, Sarit Helman, Tal Margaliot Kalifa, Sorina Grisaru-Granovsky, Orna Reichman

**Affiliations:** 1 Department of Obstetrics & Gynecology, Shaare Zedek Medical Center, Faculty of Medicine, Hebrew University of Jerusalem, Jerusalem 9190401, Israel; orenandmaayan@gmail.com (M.B.L.); majidaewida@yahoo.com (E.M.);; 2 Henrietta Szold School of Nursing, Faculty of Medicine, Hadassah Medical Center, Hebrew University, Jerusalem 9112001, Israel; amysolnica@gmail.com

**Keywords:** premature rupture of membranes, Bishop score, induction of labor, primipara

## Abstract

**Background:** Induction of labor (IOL) in nulliparas with premature rupture of membranes (PROM) and an unfavorable cervix at term poses challenges. Our study sought to investigate the impact of prostaglandin E2 (PGE2) compared to oxytocin on the duration of IOL in this specific group of parturients. **Methods**: This was retrospective matched-case study. All nulliparas with term PROM who underwent induction between January 2006 to April 2023 at Shaare Zedek Medical Center were identified. Cases induced by either PGE2 or oxytocin were matched by the following criteria: (1) time from PROM to IOL; (2) modified Bishop score prior to IOL ≤ 5; (3) newborn birthweight; and (4) vertex position. The primary outcome was time from IOL to delivery. **Results:** Ninety-five matched cases were identified. All had a modified Bishop score ≤ 5. Maternal age (26 ± 4.7 years old, *p* = 0.203) and gestational age at delivery (38.6 ± 0.6, *p* = 0.701) were similar between the groups. Matched factors including time from PROM to IOL (23.5 ± 19.2 versus 24.3 ± 21.4 *p* = 0.780), birth weight of the newborn (3111 g versus 3101 g, *p* = 0.842), and occiput anterior position (present on 98% in both groups *p* = 0.687) were similar. Time from IOL to delivery was significantly shorter by 3 h and 36 min in the group induced with oxytocin than in the group induced with PGE2 (*p* = 0.025). Within 24 h, 55 (58%) of those induced with PGE2 delivered, compared to 72 (76%) of those induced with oxytocin, (*p* = 0.033). The cesarean delivery rates [18 (19%) versus 17 (18%)], blood transfusion rates [2 (2%) versus 3 (3%)], and Apgar scores (8.8 versus 8.9) were similar between the groups (PGE2 versus oxytocin, respectively), *p* ≥ 0.387. **Conclusions:** Induction with oxytocin, among nulliparas with term PROM and an unfavorable cervix, was associated with a shorter time from IOL to delivery and a higher rate of vaginal delivery within 24 h, with no difference in short-term maternal or neonatal adverse outcomes.

## 1. Introduction

Premature rupture of membranes (PROM), occurring in 8–10% of term pregnancies, is defined as the rupture of membranes prior to the onset of labor [1]. Several studies indicate that opting for active management could potentially decrease maternal and fetal risks compared with expectant management. Active management tends to lead to a shorter duration between PROM and delivery, thereby reducing the incidence of maternal and neonatal infections [2]. The landmark Term PROM Study found that the duration from PROM to delivery was significantly shorter for women in the oxytocin group than for those in the prostaglandin (PG) E2 group, at 17.2 versus 23.0 h, respectively (*p* < 0.001) [3]. In accordance with these findings, the American College of Obstetricians and Gynecologists (ACOG) recommended intravenous oxytocin infusion over vaginal prostaglandin E (PGE) for IOL, as the latter was associated with an increased risk of chorioamnionitis, endometritis, and neonatal infection [4]. However, other studies found that vaginal PGE2 allows efficient labor induction in women with PROM and an unfavorable cervix, without a difference in maternal or neonatal complications [5,6].

Induction of labor (IOL) in nulliparas with term PROM and an unfavorable cervix poses an additional clinical challenge. Several studies have demonstrated high rates of failed induction and cesarean delivery (CD) in this population [7,8], and the best method of induction in this population has not been extensively investigated. As the Term PROM Study avoided digital examinations of parturients with PROM [3], determining the ideal IOL method for nulliparas with an unfavorable cervix remains to be determined. Some physicians opt to induce labor in these specific cases using PGE2, a common approach for nulliparas with an unfavorable cervix, regardless of PROM. 

The primary aim of this study was to investigate whether there is an association between the method of induction (oxytocin vs. cervical ripening with PGE2) and the duration from IOL to delivery among nulliparas with term PROM and an unfavorable cervix. The secondary objective was to compare the short-term maternal and neonatal complications between the two methods of induction.

## 2. Materials and Methods

A retrospective matched case study was conducted in a large tertiary university hospital, Shaare Zedek Medical Center (SZMC), Jerusalem. 

**Study population**: The obstetric characteristics of the study population are extensively described [9]. In summary, annually, SZMC conducts around 14,500 deliveries, with approximately 25% of cases being nulliparous, ~18% classified as grand multiparous (parity ≥ 6), ~12% involving CD, and ~5% utilizing vacuum deliveries. The rate of induction of labor (IOL) has increased over the past two decades, rising from 13.5% in 2006–2009 to a prevalence of 16% in 2019–2022. More than 95% of deliveries are covered by national public insurance, overseen by midwives and residents, and supervised by senior obstetricians. For the purposes of this study, all first-time mothers who underwent IOL due to PROM (ICD 658.11) from January 2006 to April 2023 were identified.

**Clinical management of term PROM**: In our department, the diagnosis of PROM involves a combination of a subjective description of rupture of membranes and objective physical examination findings, including visualization of cervical fluid pooling on speculum examination, elevated vaginal pH levels, and ultrasound confirmation of decreased amniotic fluid. In cases where the diagnosis is not definitive, we conduct the AmniSure test, which targets the placental alpha microglobulin-1 protein [1]. For most parturients experiencing term PROM, our department’s protocol includes IOL utilizing oxytocin, in accordance with the findings of the Term PROM Study. Nevertheless, there are specific situations where deviations occur: (1) occasionally, physicians opt to induce nulliparas with an unfavorable cervix using PGE2; (2) individuals undergoing a trial of labor after cesarean delivery (TOLAC) are recommended to wait for 24 h for spontaneous contractions to begin before medication intervention. The protocol for nulliparas induced with PGE2 (dinoprostone) gel includes 1–2 mg inserted into the posterior fornix. After 6 h of initiation of induction, if the cervix is dilated and effaced and contractions are inadequate, defined as fewer than 4 per 10 min, intravenous oxytocin is administered. However, if the Bishop score indicates an unfavorable cervix and inadequate contractions, a subsequent dose of PGE2 gel, 1–2 mg, is administered into the posterior fornix, for a maximum of two insertions of PGE2, followed, if indicated, by intravenous oxytocin.

**Methodology of matching**: All nulliparous patients who underwent IOL due to PROM from Jan-2006 through Apr-2023 were identified. Parturients with recorded data in the “medication of IOL” variable, which is a non-mandatory field—specifically, those whose recorded treatment involved PGE2 or oxytocin—were identified. Data including maternal age, gestational week at IOL, cervical dilatation and effacement prior to IOL, fetal head station, time from PROM to IOL, fetal head position at delivery, birth weight of the newborn, time from IOL to delivery, mode of delivery, diagnosis of chorioamnionitis, blood transfusion, Apgar score at 5’ ≤7, and admission to the neonatal intensive care unit (NICU) were retrieved from the EMR to an Excel file. To perform the matching between nulliparas induced with PGE2 and those induced with oxytocin, the data were sorted by the following criteria: (1) time from PROM to IOL, (2) modified Bishop score prior to IOL (3) newborn birthweight, and (4) fetal vertex position. The matching was performed by finding the best-fitting equal values for all four variables with priority given to time from PROM to IOL, followed by modified Bishop score, newborn birthweight, and head position. We used the accepted modification of the Bishop score that takes into account dilation, effacement, and station (each scored from 0 to 3 points), whereas a score of ≤6 is considered unfavorable [10]. For the purpose of this study, we included parturients with a modified Bishop score ≤ 5. 

**Data collection**: Data were retrieved from the electronic medical records (EMR) database regularly updated in real time by attending midwives and obstetricians during labor and delivery. Essential variables such as the time of the start of labor and parity are compulsory entries in this database. Summary notes in the EMR contain an adjusted list of pertinent diagnoses, following the International Classification of Diseases (ICD), updated by the attending physician before discharge. Consequently, it was possible to identify all first-time mothers who underwent induction of labor due to PROM (ICD 658.11) during the study years. The majority of the EMR contains fixed, obligatory fields that must be completed before the patient is transferred to the postpartum ward. Data retrieved included maternal age, gestational age at delivery, onset of labor, duration from PROM to IOL, time from IOL to delivery, mode of delivery (spontaneous vaginal, vacuum-assisted, CD), fetal head position (occiput versus posterior), maternal treatment involving packed red blood cells, newborn birth weight, Apgar score at 5 min, and the newborn’s admission ward. Additional variables, such as cervical dilation and effacement before initiating IOL and clinical chorioamnionitis, were obtained through a comprehensive review of the EMRs of all included patients in the study (M.B. and E.M). 

**Sample size**: Using data extrapolated from the Term PROM Study, we estimated the standard deviation for the oxytocin and PGE2 groups using the “range rule of thumb”, calculated as SD = (Maximum − Minimum)/4, to determine the required sample size. According to the findings of the Term PROM Study, individuals induced with oxytocin exhibited a median duration of 9.5 h (range of 2.5 to 32.9 h, from the 5th to the 95th percentile) from induction of labor (IOL) to delivery (“time to active labor” + “time to delivery”), while those induced with PGE2 had a median duration of 13.8 h (range of 2.6 to 43.7 h). If we assume that the duration from IOL to delivery remains relatively similar for first-time mothers with an unfavorable cervix, it is anticipated that IOL using oxytocin would result in a 4 h reduction in time to delivery compared to IOL with PGE2. Therefore, to detect this 4 h difference between the two medications, we calculated that a sample size of 74 in each group would be necessary, assuming a two-sided *p*-value of ≤0.05 and 80% statistical power [11]. As the majority of expectant mothers with PROM in our department undergo induction with oxytocin, the primary constraint revolved around those who were induced using PGE2. We anticipated a sizable pool of around 2000 first-time mothers with term PROM and an unfavorable cervix who would undergo induction of labor (IOL). With this estimate, we expected that at least 5% of them, roughly amounting to 100 parturients, would undergo IOL using PGE2.

**Statistics**: The validation of the data involved defining distributions and assessing missing values. Obstetric characteristics for comparison between the two groups were expressed as either proportions or means with standard deviations, depending on whether the variables were categorical or continuous, respectively. The duration from induction of labor (IOL) to delivery was compared using a Kaplan–Meier survival curve for the two groups. Statistical significance was determined with a two-sided *p*-value ≤ 0.05, employing the chi-square test or Fisher’s exact test for categorical variables.

We grappled with the approach to presenting data regarding the “modified Bishop score”. On one hand, it is a continuous variable, thus necessitating quantitative representation. Conversely, the paramount consideration lies in its clinical significance as a favorable (≥7) or unfavorable (≤6) cervix score for predicting success of IOL. Recognizing the subjective nature of the Bishop score with inherent interobserver variations, where a difference of 0.5 falls within accepted margin of error, posed a challenge, as a statistically significant finding does not necessarily align with a clinically significant finding [12]. Previous studies addressed this conflict by treating the Bishop score as a categorical variable, aligning with its clinical significance rather than its quantitative nature [3,5,6]. We opted to adopt this approach, defining an unfavorable cervix as ≤5. Additionally, we conducted a sub-analysis that included matched pairs of modified Bishop scores that were exactly matched.

All analyses were conducted utilizing IBM SPSS^®^ Statistics (version 29). 

The study was approved by the Institutional Review Board of the Shaare Zedek Medical Center (SZMC IRB 03-01-23), 28 December 2023. As this was a retrospective study, a waiver of informed consent was obtained. The manuscript is presented according to the STROBE guidelines [13].

## 3. Results

**Figure 1:**
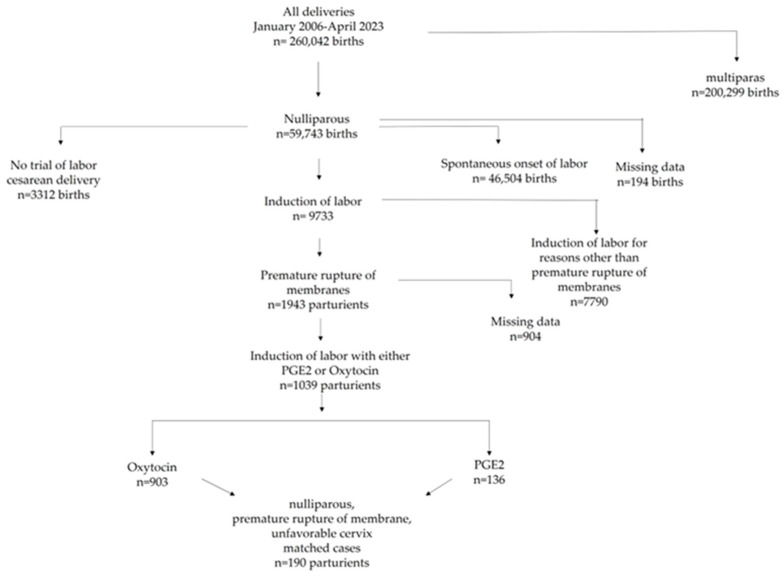
Flowchart of study population.

The initial search identified 59,743 nulliparas who gave birth at SZMC within the study period. Upon excluding mothers with spontaneous onset of labor and those scheduled for CD, a total of 9733 women who underwent IOL were identified. Among these, 1943 (20%) underwent induction due to PROM, and data regarding the administration of PGE2 or oxytocin were identified through the variable field for 1039 (53%) cases (136 [13%] PGE2 and 903 [87%] oxytocin). The final matched sample consisted of 190 nulliparas, of whom 95 underwent induction with PGE2 and 95 were induced using oxytocin, as depicted in Figure 1.

There were no statically significant differences between the two groups (PGE2 versus oxytocin) in relation to maternal age (25.9 ± 4.7 versus 26.7 ± 4.7 years old, *p* = 0.203) or gestational week at delivery (38.6 ± 0.6 in both groups, *p* = 0.701). Matched variables including time from PROM to IOL (23.5 versus 24.3 h, *p* = 0.780), birth weight of the newborn (3109 g versus 3101 g, *p* = 0.842), and occiput anterior position (present on 98% in both groups *p* = 0.687) were similar, as expected. It is noteworthy that among the matched variables, the modified Bishop score, while not clinically significant, exhibited a statistically significant difference: 2.1 versus 2.6 for PGE2 versus oxytocin, respectively, *p* = 0.004 as seen in Table 1.

Even though the duration from IOL to full dilatation did not differ significantly between the groups, the time from IOL to delivery was significantly shorter by 3 h and 36 min in the group induced with oxytocin than in the group induced with PGE2, (*p* = 0.025). CD rates (19% versus 18%), blood transfusion rates 2% versus 3%), and 5′ Apgar scores (8.9 versus 8.8, *p* ≥ 0.387) were similar in both groups. Chorioamnionitis occurred 2.5 times more in the PGE2 group (0.029), as shown in Table 2.

**Figure 2:**
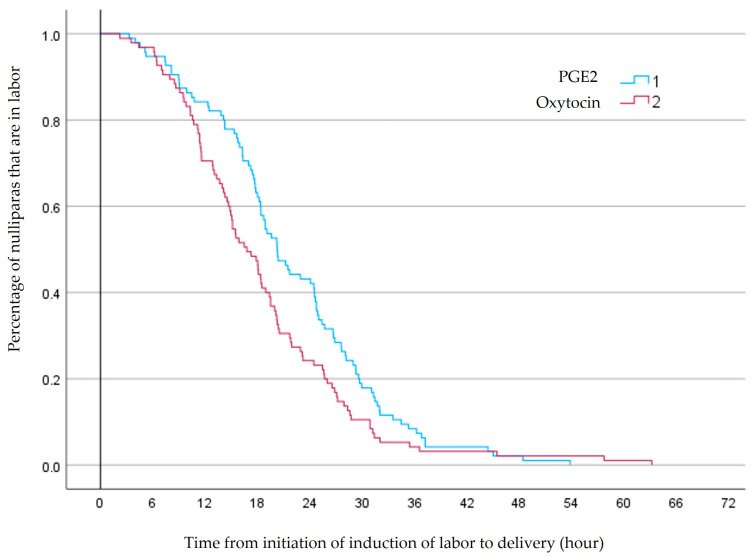
Nulliparas at term with premature rupture of membranes and a poor Bishop score, undergoing induction of labor with either prostaglandin E2 or oxytocin.

Applying the Kaplan–Meier survival curve revealed that within 24 h, 55 (58%) of those induced with PGE2 delivered, compared to 72 (76%) of those induced with oxytocin, (*p* = 0.033), Figure 2.

A sub-analysis of cases with precisely matched modified Bishop scores was conducted to ensure that the statistical difference in Bishop scores did not confound the study’s results. We omitted 30 paired cases where the modified Bishop scores did not precisely match. Consequently, the sub-analysis comprised 130 matched cases with an exact fit in the modified Bishop score, as seen by the similarity in the modified Bishop scores between the groups: 2.37 for the PGE2 group and 2.35 for the oxytocin group (*p* = 0.951), Table 3. The group induced with oxytocin experienced a significantly shorter time to delivery, with a mean time to delivery of 17.8 ± 10.1 compared to 22.4 ± 10.5 h for the PGE2 group, *p* = 0.022, Table 4.

**Figure 3:**
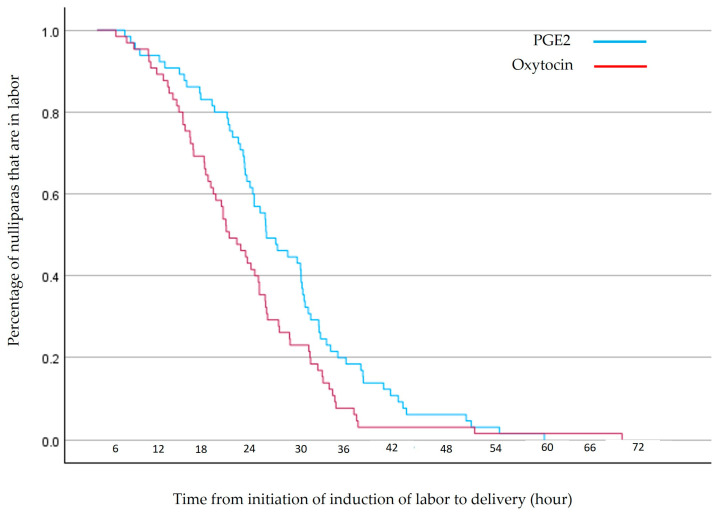
Nulliparas at term with premature rupture of membranes and precisely matched poor modified Bishop scores, undergoing induction of labor with either prostaglandin E2 or oxytocin.

Applying the Kaplan–Meier survival curve revealed that within 24 h, 37/65 (57%) of those induced with PGE2 delivered, compared to 50/65 (77%) of those induced with oxytocin, (*p* = 0.022), as shown in Figure 3.

## 4. Discussion

Based on earlier randomized controlled trials (RCTs) incorporated into a Cochrane review comparing IOL and expectant management for term PROM, there is a well-established agreement that opting for IOL significantly reduces both maternal and neonatal morbidity [2,14,15]. Included in the Cochrane review were twenty-three RCTs with 8615 women, including ten RCTs comparing intravenous oxytocin and expectant management and twelve RCTs evaluating the effect of IOL with PG (six using vaginal PGE2 and six utilizing oral, sublingual, or vaginal misoprostol (PGE1)) versus expectant management. Women who underwent IOL faced a reduced risk of maternal infectious morbidity (chorioamnionitis and/or endometritis) compared to those who received expectant management (average RR 0.49; 95% confidence interval (CI) 0.33 to 0.72; eight trials, 6864 women). In addition, women undergoing IOL experienced a shorter time from rupture of membranes to birth and had a shorter hospitalization period, and their neonates were less likely to be admitted to the neonatal intensive care unit (NICU), with a lower likelihood of definite or probable early-onset neonatal sepsis (RR 0.73; 95% CI 0.58 to 0.92; 16 trials, 7314 infants). Notably, no significant differences were observed between the IOL and to expectant management groups regarding the risk of CD, serious maternal morbidity or mortality, early-onset neonatal sepsis, or perinatal mortality [2].

Most of the trials in the Cochrane review involved both nulliparous and multiparous women, with only two trials specifically concentrating on nulliparous individuals [16,17]. Among these, one trial investigated the impact of acupuncture [16], while the other examined the effects of IOL with PGE2 compared with 24 h of observation [17]. The latter study, conducted three decades ago in Scotland, UK, involved 230 nulliparous women experiencing PROM at term with an uncomplicated singleton pregnancy. All participants had cervical dilatation < 3 cm and Bishop scores ranging from 1 to 8 at baseline. The active management of PROM with PGE2 gel demonstrated a significant reduction in the time interval from PROM to delivery. Moreover, a lower percentage of women in the PGE2 group required oxytocin augmentation (31% compared to 51%). Both management groups exhibited similarities in intrapartum analgesia, antibiotic treatment, incidence of CD, and neonatal admission to the NICU.

While the efficacy of IOL for term PROM is well established, there is less research on the preferred method for induction among nulliparas with an unfavorable cervix, particularly when comparing PGE2 and oxytocin. We have identified six studies, involving a total of 1587 primiparous women, exploring term PROM and comparing the efficacy of PGE2 or PGE1 versus oxytocin regarding “time to vaginal delivery” or “vaginal delivery within 12–24 h of labor induction.” Detailed information about these studies is presented in Table 5.

There was variability in the administration mode of the prostaglandins in the studies. Two studies utilized a slow-release PGE2 vaginal pessary [5,19], another two studies employed PGE2 pessary followed later by intravenous oxytocin infusion [6,20], and another two studies used PGE1-buccal or vaginal misoprostol [18,21]. The results across these studies were inconsistent, with some indicating a preference for PGE2 [6,20,21] and others for oxytocin [5,18,19]. In our current study, primiparous women were induced with PGE2 (dinoprostone) gel. Our findings revealed a significantly shorter time from IOL to delivery (by 3 h and 36 min) in the group induced with oxytocin than in the group induced with PGE2 (*p* = 0.025). Within 24 h, the delivery rate was 76% for oxytocin compared with 58% for PGE2 (*p* = 0.033). This corresponds with the findings reported by Kulhan, that 63% of individuals induced with oxytocin achieved vaginal delivery within 24 h, compared with 47% of those induced with PGE2 [5]. Additionally, Feret’s study observed that women receiving oxytocin had faster admission-to-delivery times than those receiving misoprostol (PGE1) (16.9 vs. 19.9 h, *p* = 0.013) [18].

The association between prolonged labor and maternal and fetal complications is firmly established, including chorioamnionitis and post-partum hemorrhage. The severity of each can necessitate interventions such as antibiotic therapy, administration of blood products, and potential admission of the newborn or mother to the intensive care unit. As such, there is an aim to prevent a prolonged labor from occurring. IOL by itself is a risk factor for prolonged labor because of the latency of the cervical ripening, which, in spontaneous onset of labor, occurs without any intervention. As such, the finding that the time from IOL with oxytocin to delivery is significantly shorter for primiparous patients with term PROM and an unfavorable cervix is clinically important. We observed that the duration to achieve full dilatation differed between women induced by PGE2 and those induced by oxytocin, with times of 18.8 h and 16.5 h, respectively. However, this difference did not reach statistical significance (*p* = 0.127). Given that the majority of CDs in our study occurred before the second stage of labor, data for the variable “time of full dilatation” was unavailable. Consequently, the sample size (n = 155) for this specific analysis may lack adequate statistical power to detect significant effects. To emphasize this limitation, we conducted a sample size calculation to determine the required sample size to detect a significant difference comparable to the observed discrepancy in time to full dilatation (18.8 h vs. 16.5 h). The calculated sample size necessary for adequate power was 514 (with 257 individuals in each group), significantly surpassing the initial sample size of 155.

We found fewer cases of chorioamnionitis in women induced with oxytocin compared with PGE2: six (6%) versus fifteen (16%), *p* = 0.029. Our findings are similar to those found by Gulersen et al., who found that cervical ripening with PGE2 was associated with an increased rate of chorioamnionitis, longer time interval from PROM to delivery, and increased NICU admissions compared with the oxytocin [19]. Since vaginal prostaglandins have been associated with a higher risk of chorioamnionitis, endometritis, and newborn infection, the American College of Obstetricians and Gynecologists (ACOG) has advocated oxytocin infusion over vaginal prostaglandin [4].

The process of IOL is complex, commencing with cervix ripening, characterized by the softening, shortening, effacement, and initial dilation of the cervix. Subsequently, myometrial contractions intensify in both tension and frequency, playing a pivotal role in advancing to active labor. Prostaglandins have a major role in the processes of cervical ripening by increasing inflammatory mediators in the cervix and inducing cervical remodeling [22,23]. Both PGE2 and PGF2a are naturally synthesized by fetal membranes and other intrauterine tissues. It is noteworthy that their concentrations are elevated in the amniotic fluid at term during labor [24,25]. This could possibly explain the findings that, during “term–PROM”, the cervix is already exposed to endogenous PGE2 washed from the amniotic fluid and the additive effect of external PGE2 is not as effective compared to all other cases of induction at term without PROM. This could probably explain the enigma in aspect of the superiority of oxytocin compared to PGE2 in IOL of an unfavorable cervix as oxytocin mainly acts on uterine muscle contraction and less on ripening the cervix. It is noteworthy that in cases of IOL other than PROM with an unfavorable cervix, PG were more effective than oxytocin in bringing about vaginal delivery within 24 h [26].

The strengths of the study included the following: (1) In the current study, 190 nulliparas were included, accounting for approximately 11% of the nulliparous population included in published research. (2) Cases and controls were matched for factors that are known to be associated with the outcome studied, “duration from IOL to delivery”, including time from PROM to IOL, modified Bishop score, fetal head position (occiput posterior versus anterior) and newborn birth weight, which decreases confounding factors. (3) The majority of variables analyzed are mandatory fields in the EMR and were, therefore, accessible.

The current study has several limitations: (1) This was a retrospective study and has the inherited limitations of retrospectively collected information. Initially, we intended to conduct a randomized controlled trial (RCT) comparing IOL with PGE2 versus oxytocin for the specific group of term PROM nulliparas with unfavorable cervices. However, barriers such as the high workload in the delivery room and ethical considerations—where initiating the study for patients recruited for the study over those that were waiting for IOL for other reasons seemed unethical and prevented the commencement of the study. Therefore, we opted for a retrospective matched case study of term PROM nulliparas with an unfavorable cervix, comparing those induced with PGE2 with those induced with oxytocin. (2) We do not have data regarding the BMI of parturients as this information was not routinely collected during these years. The study had a relatively small sample size for the investigation of secondary outcomes such as maternal and neonatal complications. (3) Single-center studies, due to their nature, tend to be homogeneous, unlike multicenter studies that may exhibit variations in obstetric management and treatment protocols, potentially impacting the external validity of the study.

In summary, based on our findings, for nulliparas experiencing term PROM with an unfavorable cervix, initiating IOL with oxytocin may result in a reduced duration from the start of induction to delivery. However, considering the inconsistent findings observed across other studies, the retrospective methodology employed in all but one and the use of different formulations of prostaglandins for induction, it is imperative for future randomized controlled trials (RCTs) be conducted. This approach would enhance the rigor and reliability of the study, providing more robust evidence and minimizing potential biases associated with retrospective methodologies and diverse induction agents.

## Figures and Tables

**Table 1:** The obstetric profiles of 190 matched* nulliparas at term with premature rupture of membranes and an unfavorable cervix who underwent labor induction with either PGE2 or oxytocin.

	PGE2 n = 95	Oxytocin n = 95	*p* Value
Maternal age	25.9 ± 4.7	26.7 ± 4.5	0.227
Gestational week	38.6 ± 0.9	38.6 ± 0.9	0.707
Modified Bishop score ≤ 5	95 (100%)	95 (100%)	1.000
Cervical dilatation (cm)	0.77 ± 0.6	1.04 ± 0.8	0.009
Modified Bishop score	2.07 ± 1.3	2.64 ± 1.3	0.004
Time from PROM to IOL (hours)	23.5 ± 19.2	24.3 ± 21.4	0.925
Vertex position occipital anterior	93 (98%)	94 (98.9%)	0.500
Birth weight of newborn (grams)	3109 ± 392	3101 ± 334	0.879

* Cases were matched for (1) modified Bishop score, (2) duration from membrane rupture to the start of labor induction, (3) fetal head presentation during delivery, and (4) the newborn’s birth weight.

**Table 2:** Short-term maternal and neonatal outcomes of 190 matched* nulliparas at term with premature rupture of membranes and an unfavorable cervix who underwent labor induction with either PGE2 or oxytocin.

	PGE2 n = 95	Oxytocin n = 95	*p* Value
Time from IOL to full dilatation (hours)	18.8± 9.5	16.5 ± 9.1	0.127
Length of second stage (hours)	1.7± 1.1	1.6± 1.0	0.582
Time from IOL to delivery (hours)	21.8± 10.0	18.5 ± 10.25	0.025
Mode of delivery			
Spontaneous vaginal delivery	59 (62%)	65 (68%)	0.387
Assisted vaginal delivery	19 (20%)	12 (13%)	
Cesarean delivery	17 (18%)	18 (19%)	
Chorioamnionitis	15 (16%)	6 (6%)	0.029
Blood transfusion	3 (3%)	2 (2%)	0.505
Apgar score at ′ 5 ≤ 7	3 (3%)	2 (2%)	0.571
Admission to NICU **	1 (1%)	4 (4%)	0.187

* Cases were matched for (1) modified Bishop score, (2) duration from membrane rupture to the start of labor induction, (3) fetal head presentation during delivery, and (4) the newborn’s birth weight. ** NICU-Neonatal Intensive Care Unit.

**Table 3:** The obstetric profiles of 130 matched nulliparas at term with premature rupture of membranes and precisely matched unfavorable Bishop scores who underwent labor induction with either PGE2 or oxytocin.

	PGE2 n = 65	Oxytocin n = 65	*p* Value
Maternal age	26.3 ± 4.8	26.8 ± 4.7	0. 477
Gestational week	38.9 ± 0.9	38.9 ± 0.9	0.787
Cervical dilatation (cm)	0.84	1.09	0.033
Modified Bishop score	2.37 ± 1.4	2.35 ± 1.4	0.951
Time from PROM to IOL (hours)	23.6 ± 20.6	24.5 ± 23.4	0.810
Vertex position occipital anterior	65 (100%)	64 (98.5%)	0.500
Birth weight of newborn (grams)	3127 ± 432	3122 ± 392	0.945

**Table 4:** Short-term maternal and neonatal outcomes of 130 matched nulliparas at term with premature rupture of membranes and precisely matched unfavorable Bishop scores who underwent labor induction with either PGE2 or oxytocin.

	PGE2 n = 65	Oxytocin n = 65	*p* Value
Time from IOL to full dilatation (hours)	19.2 ± 9.9	16.4 ± 9.8	0.157
Time from IOL to delivery (hours)	22.4 ± 10.5	17.9 ± 10.1	0.014
Mode of delivery			
Spontaneous vaginal delivery	39 (60%)	44 (68%)	0.273
Assisted vaginal delivery	15 (23%)	8 (12%)	
Cesarean delivery	11 (17%)	13 (20%)	
Chorioamnionitis	11 (17%)	4 (6%)	0.049
Blood transfusion	3 (4.6%)	2 (3%)	0.505
Apgar score ′5 ≤ 7	2 (3%)	2 (3%)	0.721
Admission to NICU **	1 (1.5%)	4 (6.2%)	0.183

** NICU-Neonatal Intensive Care Unit.

**Table 5:** Comparative trials investigating either prostaglandin E2 or E1 versus oxytocin for induction of labor in nulliparous women with an unfavorable cervix.

Study Primary Author (ref#)	Method of Study	Sample Size (Primiparous)	Primary Outcome	Result
Kulhan [5]	Retrospective Primiparous only Oxytocin versus PGE2 vaginal pessary, slow-release	224	Vaginal delivery within 24 h of labor induction	Oxytocin 72/112 (64.3%) vs. PGE2 53/112 (47.3%), *p* = 0.023
Güngördük [6]	RCT Mixed parity Oxytocin versus PGE2 pessary followed 6 h later by intravenous oxytocin infusion	238	Vaginal delivery within 24 h of labor induction	PGE2 72% vs. oxytocin 55%; relative risk, 1.30; 95% confidence interval, 1.07–1.59; *p* = 0.007)
Feret [18]	Retrospective Primiparous only Oxytocin versus buccal misoprostol	130	Time from admission to delivery	Women receiving oxytocin had faster admission-to-delivery times than women receiving misoprostol (16.9 vs. 19.9 h, *p* = 0.013)
Gulersen [19]	Retrospective Primiparous only Oxytocin versus PGE2 vaginal pessary, slow-release	275	Time interval from PROM to delivery/chorioamnionitis/neonatal intensive care unit (NICU) admissions	Time interval from PROM to delivery PGE2 24.4 vs. oxytocin 17.9 h; aHR 0.56, *p* ≤ 0.0001 Chorioamnionitis PGE2 18.1% vs. oxytocin 6.1%; aOR 4.14, *p* = 0.001 NICU PGE2 20.2% vs. oxytocin 9.9%; aOR 2.4, *p* = 0.02
Liu [20]	Retrospective, primiparous only, PGE2 pessary followed by oxytocin infusion versus oxytocin	205	Vaginal delivery within 12 h and total vaginal delivery	vaginal delivery within 12 h and total vaginal delivery were higher in the PGE2 group (28.4% vs. 7.8%, *p* = 0.0001; 79.4% vs. 62.1%, *p* = 0.009, respectively)
Zhang [21]	Retrospective, mixed parity, oxytocin versus vaginal misoprostol	515	Vaginal delivery within 24 h of labor induction and cesarean delivery rate	More nulliparas in the misoprostol group achieved vaginal delivery within 24 h than in the oxytocin group (60.5% vs. 45.4%, *p* = 0.001), and there was a lower cesarean delivery rate (12.6% vs. 27.5%, *p* < 0.001)
Bas. current study	Retrospective, matched cases and controls Primiparous only Oxytocin versus vaginal PGE2 gel	190	Time from induction to delivery/chorioamnionitis/	Within 24 h delivered, PGE2 55(58%) compared to oxytocin 72 (76%), (*p* = 0.033) Chorioamnionitis PGE2 16% vs. oxytocin 6%, *p* = 0.029

## Data Availability

Data are available upon reasonable request from the corresponding author.
